# Recent Advances in the Role of Fibroblast Growth Factors in Hair Follicle Growth

**DOI:** 10.3390/biom15081198

**Published:** 2025-08-20

**Authors:** Junchao Wang, Lusheng Wang, Shuang Gao, Xiaokun Li

**Affiliations:** 1 School of Pharmaceutical Sciences, Wenzhou Medical University, Wenzhou 325035, China; junchaowang@wmu.edu.cn (J.W.); gaoshuangphu@wmu.edu.cn (S.G.); 2 National Clinical Research Center for Ocular Diseases, Eye Hospital, Wenzhou Medical University, Wenzhou 325027, China; wanglusheng@eye.ac.cn

**Keywords:** hair follicle, FGF/FGFRs, alopecia, therapy

## Abstract

Hair follicles are essential to hair formation and cyclic regeneration, experiencing growth and degeneration, and quiescence phases involving complex signaling pathways. Among these, fibroblast growth factors (FGFs) play a critical role in follicular morphogenesis, but the role of FGF receptor signaling in hair follicle development remains underexplored. Current treatments for hair loss, such as medical, surgical, light-based, and nutraceutical interventions, are often expensive, require long-term commitment, and are associated with substantial side effects. This review discusses the mechanisms and biological functions of the FGF signaling pathway within the hair follicle growth cycle, providing an overview of how these elements influence hair follicle dynamics and the pathogenesis of alopecia. Manipulating the FGF signaling pathway could offer new therapeutic options for androgenetic alopecia and other hair loss conditions, potentially exceeding current treatment modalities in efficacy and safety.

## 1. Introduction

Various physiological factors and signaling pathways within the human body influence hair growth. At the heart of this process lies the hair follicle, a dynamic and highly organized structure that undergoes repetitive cycles of growth, regression, and rest, known as the anagen, catagen, and telogen phases, respectively. Many biological signals control these phases, ensuring hair follicles’ continued renewal and proper functioning. Among the key regulators are fibroblast growth factors (FGFs) and their receptors (FGFRs), which have been recognized for their critical roles in hair follicle development and cycling [1,2]. Despite considerable advances in understanding FGF involvement in hair biology, the literature reveals a gap in comprehensive, mechanism-focused understandings of how FGF pathways can be manipulated to counteract hair loss conditions. This review discusses the role of FGF pathways and the underlying molecular mechanisms in promoting hair growth and treating alopecia.

FGF therapies demonstrate several advantages over conventional treatments like minoxidil and finasteride. Unlike these commercial options, which often have limited efficacy and side effects, FGFs specifically target hair follicle regeneration through multiple mechanisms [3]. For instance, FGF12 promotes hair growth by inducing the anagen phase and regulating growth factors like PDGF-CC and HB-EGF, while rhFGF9 activates the TGF-β/BMP/Smad signaling pathway [4,5]. This targeted approach contrasts with minoxidil’s nonspecific vasodilation or finasteride’s systemic DHT reduction [6]. Compared to surgical interventions, FGF treatments offer non-invasive alternatives with regenerative potential. Studies show FGF formulations can maintain hair bulb integrity and delay catagen transition, achieving results comparable to conventional products [7]. The FGF family’s natural role in hair cycle regulation provides physiological advantages over synthetic drugs, potentially reducing adverse effects. Emerging evidence suggests FGF therapies may complement other advanced treatments. For example, they share mechanistic similarities with Platelet-Rich Plasma (which contains endogenous growth factors) [8,9], but offer more standardized formulations than autologous preparations. While cell-based therapies remain clinically immature, FGF proteins present a more immediately viable biological approach [10].

Compared with emerging treatment methods, FGF treatment still has many advantages. While PRP contains multiple growth factors (e.g., PDGF, VEGF) and shows efficacy in wound healing and hair growth, its composition varies between individuals, leading to inconsistent outcomes [11,12]. PRP-derived exosomes improve standardization but still rely on donor variability and complex isolation processes. In contrast, FGF offers precise dosing and targeted action without these limitations [13]. Exosome therapy (e.g., from PRP or stem cells) promotes hair regeneration by modulating dermal papilla cells and stem cell activation [14,15]. However, exosome production lacks standardization, and their mechanisms are indirect compared to FGF’s direct growth factor signaling [16,17]. Exosomes also face challenges in sustained delivery, often requiring biomaterial carriers (e.g., microneedle patches) for efficacy. Microneedling primarily acts as a physical enhancer of drug delivery; its standalone effects are modest, often requiring adjunctive treatments to achieve clinically meaningful results [18]. In contrast, recombinant FGF therapies provide targeted signaling. FGF-based interventions have demonstrated strong efficacy in wound healing and tissue regeneration, ranking highly for healing time and ulcer area reduction alongside PRP and PDGF [11]. Despite its advantages, FGF’s short half-life may necessitate repeated applications or advanced delivery systems (e.g., hydrogels) to prolong activity [13]. In contrast, PRP and exosomes provide sustained growth factor release but with less predictability.

In summary, FGF therapies offer superior standardization, direct efficacy, and safety over other therapies, though delivery optimization remains a challenge. It also exhibits a favorable safety profile with minimal adverse effects, making it a reliable option for clinical applications.

## 2. Hair Follicle

### 2.1. Hair Follicle Formation

The hair follicle, the primary skin appendage, originates in the ectoderm. It functions as a stem cell reservoir and a hair shaft factory, contributing to restating its surrounding skin microenvironment, including skin innervation and vascularization. Hair follicle morphogenesis and regeneration are driven by the close interaction between the epithelial (epidermal stem cells [Epi-SCs]) and the mesenchymal components (dermal papilla [DP]), a process known as the epithelial-mesenchymal interaction (EMI). Hair follicle morphogenesis occurs in three main developmental stages: induction, organogenesis, and cell differentiation. During the induction stage (stage 1), localized thickening of the surface epithelial cells forms the hair follicle precursor. Organogenesis (stages 2 and 3) begins with the aggregation of mesenchymal cells beneath the precursor, leading to the downward growth of epithelial cells and the formation of a hair bud. The hair bud multiplies and invaginates deeper into the dermis (stage 4, nail stage). Cell differentiation begins when the downward-growing epithelial cells reach their final length (stage 5, bulbous peg stage). In this phase (stages 5–8), the edges of the downward-growing follicular epithelium gradually envelop the mesenchymal aggregates (bulbar embolus stage), forming the dermal papilla. Signals from the dermal papilla induce adjacent epithelial cells to differentiate into the inner root sheath (IRS). This provides a mold for the developing hair shaft, which elongates and eventually penetrates the epidermis. The formation of the funnel cavity and its opening occurs during stages 6–8 [19].

### 2.2. Structure and Morphology of Hair Follicles

**Figure 1:**
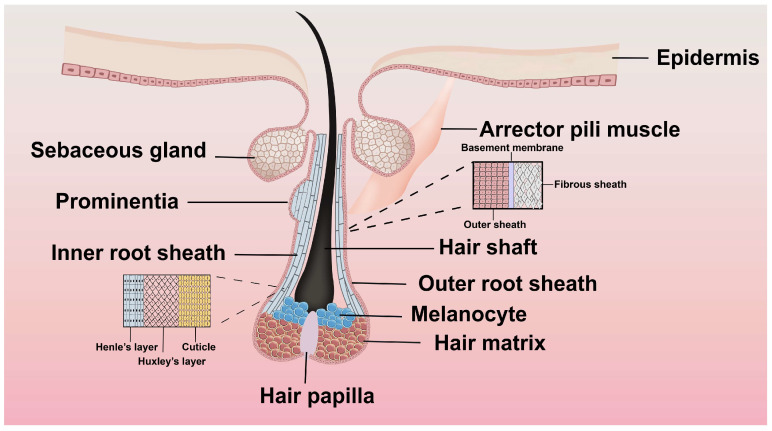
Structure of a hair follicle. The hair follicle is a tubular structure that includes several layers: the connective tissue sheath, the outer root sheath, the inner root sheath, and the central hair shaft.

The hair follicles are anatomically segmented into the infundibulum, isthmus, bulge, and bulb from top to bottom. The infundibulum on the skin’s surface connects to the sebaceous duct at its lower end. The isthmus widens at its lower portion into a protuberance, where the arrector pili muscle attaches [20]. During the growth phase, the hair follicle’s lower end forms a bulbous structure known as the hair bulb, at the center of which the dermal papilla, fusiform in shape, is situated. Comprising dermal papilla cells (DPCs) from the interstitial area, the DP’s primary role is to induce and sustain the growth and differentiation of hair follicle epithelial cells and to initiate hair follicle morphogenesis. The inner and outer root sheaths encase the hair shaft. The inner sheath consists of the sheath cuticle, the Huxley layer, and the Henle layer, while the outer sheath comprises multiple cellular layers that envelope the inner sheath and the hair shaft. Surrounding the entire structure is the connective tissue sheath, made of fibroblasts, which isolates the hair follicle from the surrounding dermis [21]. Figure 1 shows the structural layout of the hair follicle. The diagram clearly shows the sebaceous gland associated with the upper follicle, the arrector pili muscle attaching laterally, the concentric layers of the inner and outer root sheaths surrounding the hair shaft, the location of melanocytes within the matrix adjacent to the papilla, and the critical inductive role of the dermal hair papilla at the follicle base.

Morphogenesis of hair follicles begins in the embryonic stage and involves significant interactions among the Wingless/Integrated signaling pathway (Wnt), sonic hedgehog (Shh), Notch, and bone morphogenetic protein (BMP) signaling pathways [22]. This process is divided into the induction, organogenesis, and cell differentiation phases. The induction phase is characterized by Wnt-mediated signaling in mesenchymal cells, which directs the thickening of the epithelial layer into plaques. During organogenesis, a dynamic exchange of signals occurs, prompting epithelial cells to instruct underlying dermal cells to proliferate and form dermal condensates [23]. These, in turn, drive epithelial proliferation downward into the dermis. In the cell differentiation phase, dermal condensates are encapsulated by follicular epithelial cells to form discrete dermal papillae. This illustrates the role of ectoderm in shaping the hair follicle through morphogens and growth factors [24,25].

### 2.3. Classification of Hair Follicles

Hair follicles are categorized into two primary types: primary and secondary hair follicles [26,27]. Primary hair follicles, which form first, are followed by the development of secondary hair follicles and their branches. The morphogenesis and development of primary hair follicles are crucial to determine the yield and quality of these structures [28]. In examinations of skin hair follicles, the primary follicles feature a large hair bulb located deep within the dermis. A thick diameter and a medullated hair shaft characterize these follicles. In contrast, secondary hair follicles have a smaller hair bulb located in the superficial layer of the skin, have a narrower diameter, and lack a medulla in the hair shaft. Primary and secondary hair follicles share the same fundamental structure despite these differences. Research indicates that all hair follicles originate from hair follicle stem cells in the bulge area near the skin’s surface [29].

### 2.4. Periodic Changes in Hair Follicles

**Figure 2:**
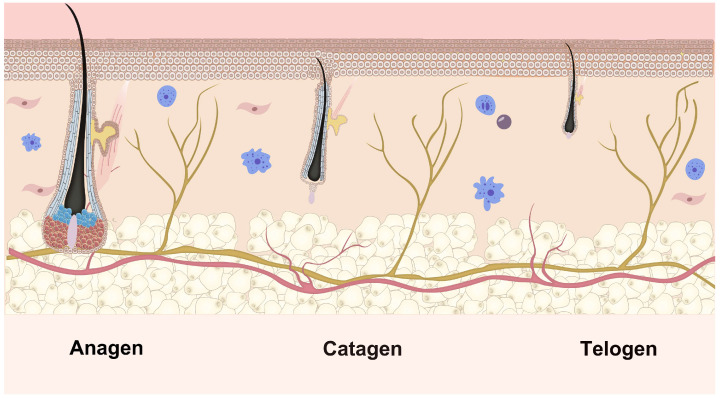
Hair follicle growth cycle. The continuous cyclical regeneration of the hair follicle maintains hair growth. The upper segment above the arrector pili muscle attachment remains unchanged and consistent. The lower segment undergoes cyclical transformations through the hair cycle’s growth, regression, and rest phases. These changes in the lower segment categorize the hair growth cycle into three stages: growth, degeneration, and quiescence [34].

Hair growth is sustained by the cyclical regeneration of hair follicles, which undergo repeated cycles of growth, degeneration, and rest throughout their development. The growth phase is categorized into six stages, from I to VI. Conversely, hair degeneration comprises eight stages, from regression stage I to regression stage VIII. Various signaling mechanisms govern the transition from one stage to the next during the growth phase. In this phase, the asymmetric division renews the progenitor cell compartment, supplying a vertical flux of progeny cells. These cells undergo limited divisions and differentiate to form the inner root sheath (IRS) components and the hair shaft. Adding new components at the inner layer’s base stimulates the hair shaft’s growth, facilitating its emergence from the skin [30]. At the end of the growth phase, proliferation within the hair matrix halts, ushering in the degeneration phase. During this phase, apoptosis is initiated in the stroma surrounding DP and progresses upward, resulting in shortening and degradation of the follicle [31]. The quiescent phase follows, during which the DP recedes along the degraded epithelial chain and settles beneath the permanent portion of the follicle [32]. The entire growth cycle of the follicle not only facilitates self-renewal but also regulates hair growth and shedding. The transition through the follicle cycle is based on a dynamic equilibrium between various regulators that promote and inhibit hair follicle growth [33]. This complete growth cycle is shown in Figure 2. This schematic illustrates the three distinct phases of the hair follicle growth cycle. Anagen is a stage of rapid growth, characterized by rapid hair shaft production. Catagen is a brief transitional phase involving follicular regression and detachment from the dermal papilla. This cyclical process is essential for continuous hair renewal. Telogen is a resting phase, culminating in hair shedding and preparation for the next anagen phase.

The process of hair growth itself is intricately coordinated at the cellular level. It begins with the activation of hair follicle stem cells (HFSCs), which remain dormant in the bulge region of the follicle during the telogen phase [35]. Upon transitioning into anagen, these stem cells are activated and migrate towards the hair matrix, where they interact with DPCs. This interaction triggers the proliferation and differentiation of hair matrix cells (HMCs), which are responsible for synthesizing keratin and forming the hair shaft [36]. DPCs, positioned at the base of the follicle, serve as the “command center” by secreting signaling molecules that regulate the proliferation and differentiation of HMCs [37]. As the hair matrix cells differentiate, they begin to align vertically, a process that is driven by molecular signaling pathways such as ERK1/2 and p38 MAPK, which coordinate cell migration and keratin deposition [38]. This cellular alignment physically propels the growing hair shaft upwards, out of the follicle, towards the skin surface. Throughout this process, feedback mechanisms, such as the modulation of soluble epoxide hydrolase (SEH) activity, help sustain the anagen phase and ensure the continuous growth of hair follicles [39]. If this delicate balance is disrupted, as seen in aging or pathological conditions, hair growth can be impaired, leading to hair loss [40,41]. Thus, the process of hair growth is a dynamic interplay of cellular activation, molecular signaling, and physical movement, ensuring that hair follicles continuously regenerate and produce new hair fibers throughout the hair cycle. This coordination is crucial for maintaining hair health and understanding the mechanisms of hair diseases.

### 2.5. Cell Toxicity-Induced Hair Loss Mechanisms

Cell toxicity, caused by chemotherapy drugs, radiation, and environmental toxins, significantly disrupts hair follicle function and induces hair loss. Chemotherapy drugs, such as cisplatin and cyclophosphamide, target rapidly dividing cells, including hair follicle keratinocytes. Cisplatin, for example, accumulates in follicular mitochondria, causing mitochondrial dysfunction and cell death, leading to hair follicle damage and subsequent hair loss [42]. Chemotherapy-induced alopecia (CIA) is a well-known side effect, causing significant hair follicle damage and hair thinning [43]. Radiation therapy induces apoptosis in transit-amplifying cells in hair follicles, leading to widespread cell death, particularly in the early stages of treatment. This damages follicular homeostasis, resulting in radiation-induced alopecia [44]. Environmental toxins, such as pollutants and UV radiation, generate free radicals that cause oxidative stress and inflammation, disrupting follicular function and accelerating follicle degradation [45]. Oxidative stress is a key factor in hair loss, inducing DNA damage and cell death, further compromising hair follicle health [46].

Cell toxicity also affects the hair follicle cycle, accelerating the transition from the anagen phase to the catagen phase. Apoptotic signals, such as apoptosis-inducing factor (AIF) nuclear translocation and poly [ADP-ribose] polymerase 1 (PARP1) upregulation, trigger cell death and follicle shedding during this transition [47]. Oxidative stress and ROS drive follicle cycle alterations, exacerbating cell loss through DNA damage. In some cases, cytotoxicity suppresses hair follicle stem cell activity, reducing hair regeneration and contributing to long-term hair loss [48]. Ultimately, apoptosis and autophagy are central mechanisms in cytotoxic-induced hair follicle degradation. Apoptosis is induced by chemotherapy drugs and radiation through mitochondrial dysfunction and caspase activation, leading to follicular cell loss [49]. Dysregulated autophagy can also contribute to cell death in response to cytotoxic stress [50]. These mechanisms collectively result in irreversible follicular degeneration and hair loss.

## 3. Expression and Distribution of FGFs and Their Receptors in Hair Follicles

### 3.1. Expression and Distribution of FGFs in Hair Follicles

**Figure 3:**
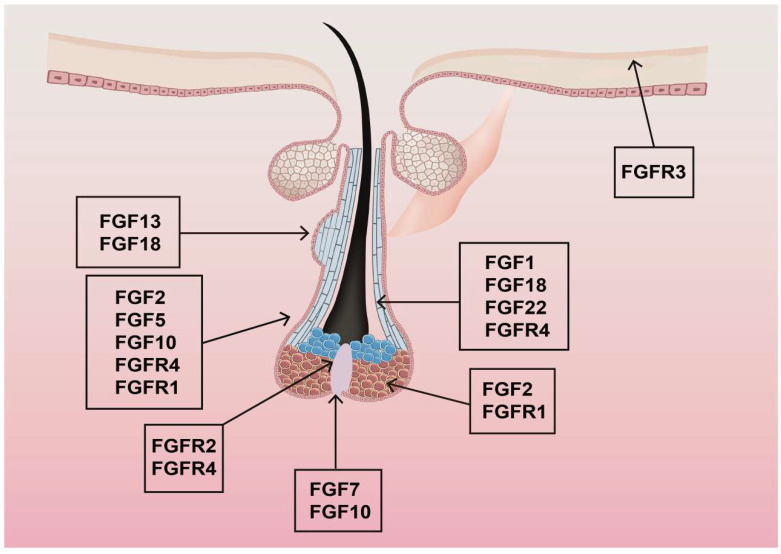
Distribution of different FGF transcripts in a hair follicle. mRNAs for FGFs and their receptors are in various segments of the hair follicle and have distinct functions.

In both mice and humans, 22 FGF ligands have been identified. These FGFs are categorized into six subfamilies based on sequence homology and phylogenetic relationships. There are five paracrine subfamilies and one endocrine subfamily (FGF1 and FGF2). Paracrine subfamilies include the FGF4 subfamily (FGF4, FGF5, FGF6), the FGF7 subfamily (FGF3, FGF7, FGF10, FGF22), the FGF8 subfamily (FGF8, FGF17, FGF18), the FGF9 subfamily (FGF9, FGF16, FGF20), and the FGF19 subfamily (FGF19, FGF21, FGF23), which is involved in endocrine-dependent signaling [51]. The expression levels of these FGFs in hair follicles vary. FGF1, FGF5, FGF7, FGF10, FGF13, FGF18, and FGF22 show high expression levels. In contrast, FGF2, FGF3, FGF6, FGF9, FGF11, FGF20, and FGF21 exhibit moderate expression. FGF4, FGF8, FGF12, FGF14, FGF15, FGF16, FGF17, and FGF23 are expressed at deficient levels or are non-detectable. Table 1 and Figure 3 detail and illustrate the distribution of mRNAs for different FGFs in various hair follicle growth cycles. Figure 3 illustrates the spatial expression patterns of key fibroblast growth factors (FGFs) and their receptors (FGFRs) within the hair follicle microenvironment. Multiple FGF ligands—including FGF1, FGF2, FGF5, FGF7, FGF10, FGF13, FGF18, and FGF22—are localized to distinct compartments, suggesting stage-specific roles in follicular development, cycling, and differentiation. Concurrently, receptors FGFR1, FGFR2, FGFR3, and FGFR4 exhibit overlapping yet compartmentalized expression, highlighting potential autocrine/paracrine signaling axes.

### 3.2. Expression and Distribution of FGF Receptors in Hair Follicles

As single transmembrane proteins, FGF receptors (FGFRs) are known for their versatile roles in cell signaling due to their ability to bind multiple ligands [51]. FGFR1 and FGFR3 are highly expressed in hair follicles, while FGFR4 shows low expression. FGFR1 is predominantly found during growth phase VI and the regression phase. Both FGFR2 and FGFR3 reach peak expression during growth phase VI. This varied expression of FGF family members and their receptors at different stages of the hair follicle growth cycle suggests distinct impacts on hair follicle development.

In situ RNA hybridization analysis has revealed specific expression patterns: FGFR1 is present in dermal papilla cells and transiently in the outer root sheath (ORS) cells in the lower part of the hair follicle during growth phases. FGFR2 is in hair progenitor cells near the dermal papilla. FGFR3 is found in the cell layer on the periphery of the hair bulb, continuous with the hair shaft cuticle and the IRS, with its expression potentially extending to the keratinization zone at the top of the hair bulb. FGFR4 is expressed in inner and outer root sheath cells surrounding the hair bulb and in the lower part of the hair follicle. However, these receptors are not expressed during the late stages of follicle degeneration or the rest period [65,66].

## 4. The Regulatory Role of FGFs and FGFRs in Hair Follicle Cycles

The transition of the hair follicle cycle is governed by a balance between regulatory factors that promote and those that inhibit hair growth. Table 2 outlines the functions of FGFs within the hair follicle, as identified in recent studies. FGF7 and FGF10, which can bind to the abundantly expressed FGFR2 in hair follicle stromal cells, stimulate stromal cell proliferation and initiate the hair follicle growth period. FGF20 and FGF21 are critical in inducing cell differentiation in the basal layer, promoting hair follicle formation and localization. As hair follicle stem cells continually differentiate, FGF22 expression peaks in growth phase VI, corresponding to the maximum length of the hair follicles. FGF18 has been shown to induce DNA synthesis in various cells, including human hair follicles, DPCs, dermal fibroblasts, and epidermal keratinocytes. It facilitates the transition from resting to the growth phase and enhances overall hair follicle growth [67]. FGF23 interacts with FGFR 1c-α-Klotho, FGFR 3c-α-Klotho, and FGFR 4-α-Klotho complexes. Meanwhile, FGF19 primarily activates FGFR 1c-β-Klotho (KLB) and FGFR 4-KLB, while FGF21 predominantly stimulates the FGFR 1c-KLB complex [51].

FGFR deficiency can inhibit growth, and each receptor plays a distinct role in parts of the hair follicles. For instance, FGFR2 deficiency may lead to delayed hair follicle development and severe epidermal hypoplasia. FGFR3 is suggested to play a catalytic role in root sheath differentiation and hair follicle growth [52]. Although FGFR4 is expressed at deficient levels in hair follicles and is less studied in hair cycle regulation, it also regulates tumor development and progression.

## 5. Recent Research on FGFs Detected During Growth

### 5.1. FGFs That Are Found During Growth and Promote Hair Growth

Several hair-promoting FGFs have been identified during the hair growth phase. FGF7, known as keratinocyte growth factor 1 (KGF1), promotes keratinocyte growth and is prominently expressed in various hair follicle components, including DPCs, IRS cells, hair follicle mesenchyme, and epithelium. FGF7 is expressed at elevated levels during the early growth phase, which induces and maintains hair follicle growth. It also enhances hair shaft growth and elongation in xenograft-cultured hair follicles and improves the survival of hair follicles following chemotherapy and other cytotoxic injuries [76].

The absence of the epidermal growth factor receptor (EGFR) can facilitate microbial growth and the penetration of microorganisms into hair follicles through open follicles during hair germination, leading to chronic folliculitis. Preventive treatment with FGF7 has been shown to restore extracellular signal-regulated kinase (ERK) signaling and effectively prevent microbial invasion without EGFR, alleviating follicle inflammation [24,77].

BeauTop (BT), a health food supplement, has demonstrated efficacy in promoting hair growth in mice by increasing FGF7 protein levels [78]. Its active ingredients include *Ginseng radix*, *Astragali radix*, *Radix Angelicae sinensis*, *Ligustri fructus*, *Rehmannia glutinosa*, and *Eclipta prostrata* (Linn). FGF7 and Noggin are crucial in initiating new hair growth cycles [79,80]. In the initial growth cycle, FGF7 is expressed mainly in hair follicles’ root sheath and epidermis, significantly influencing early follicle development and induction of new growth cycles [81]. Results found that all members of the FGF7 subfamily were among the key DEGs screened; the differential expression of FGF7 and FGF10 and their receptors, FGFR1/FGFR2, was verified between ESG placode-containing skin and HF placode-containing skin. In vivo and in vitro Matrigel plug models showed that both FGF7 and FGF10 promoted fate transition of human epidermal cell-derived organoids to ESG phenotype organoids, FGF7 and FGF10 had a synergistic effect, and they mainly function through the FGFR1/2-MEK1/2-ERK1/2 pathway [77].

FGF10, also known as KGF2, is a member of the FGF7 subfamily crucial for hair follicle development. Mice knockout studies have shown that the absence of the *Fgf10* gene leads to hair follicle hypoplasia and dysplasia [82]. In secondary hair follicles, *miR-184* within DPCs (SHF-DPCs) influences hair growth by modulating FGF10 levels. Knockdown of *miR-184* improves FGF10 expression, increases the proportion of cells in the S phase, promotes cell proliferation, reduces apoptosis, and impacts cashmere development [83]. The coding region sequence (CDS) of the *Fgf10* gene in Chinese Merino sheep spans 696 base pairs and encodes a protein of 231 amino acids. This sequence is highly conserved between species and is expressed in skin tissues of sheep hair follicles at various developmental stages, highlighting the significant biological role of FGF10 in the growth and development of sheep hair follicles [28,84].

Applying safflower oil body human fibroblast growth factor 10 (SOB-hFGF10) SOB-hFGF10 to androgenetic alopecia (AGA) mice has significantly improved hair regeneration and growth. This treatment also upregulates the expression of hair growth-related proteins in these mice, presenting an effect similar to minoxidil [85]. However, SOB-hFGF10 offers advantages over minoxidil, including better penetration of the follicle through the sebaceous pathway and superior biosafety and targeting profiles [86,87,88].

FGF12, structurally similar to FGF1, is pivotal in stimulating cell proliferation and hair growth. It regulates critical signaling pathways, including mitogen-activated protein kinase (MAPK), phosphoinositide 3-kinase (PI-3)/Akt, and Rat sarcoma (Ras) [28,89]. Predominantly expressed in ORS cells, FGF12 shows high expression levels during the growth phase of hair follicles [4]. In murine models, *Fgf12* gene knockdown delays the transition from resting to growth stages, reducing hair length in tentacular hair follicles and inhibiting ORS cell proliferation and migration. Conversely, *Fgf12* overexpression enhances ORS cell migration and facilitates animal models’ transition from resting to growth phases [90]. Additionally, FGF12 overexpression upregulates the expression of growth factors such as platelet-derived growth factor (PDGF)-CC, midkine (MDK), and heparin-binding EGF-like growth factor (HB-EGF), which promote hair growth [91]. FGF12 interacts with the Wnt/β-catenin pathway, which is essential for hair follicle morphogenesis, stem cell activation, and regeneration. Wnt signaling induces dermal papilla formation and governs hair follicle stem cell fate [4,92,93].

The *Fgf22* gene, which undergoes pseudogenization, is enriched in hair follicles and plays a significant role in their formation and maturation [94]. In mice, *Fgf22* expression varies throughout the growth cycle, peaking in late growth and early degeneration phases within ORS, epidermis, and sebaceous glands of hair follicles [86]. This expression pattern suggests the critical role of *Fgf22* in inducing hair follicle degeneration. Moreover, FGF22 is believed to promote the maturation of hair follicles during the foundational stages of their development [95].

The *Fgf22* gene, enriched in hair follicles, undergoes pseudogenization. In mice, *Fgf22* expression varies throughout the growth cycle, peaking in late growth and early degeneration phases within the hair follicles’ ORS, epidermis, and sebaceous glands. Expression peaks during the late growth and early degeneration phases, in contrast to lower levels observed in other phases. It then decreases as the hair follicles transition to the quiescent phase. These patterns indicate a potential key role for *Fgf22* in promoting hair follicle degeneration. Additionally, FGF22 is crucial for the formation and maturation of hair follicles, potentially enhancing their maturation during the initial stages of development [96].

In the late anagen, FGF22 primarily signals to precortical keratinocytes and hair matrix keratinocytes within the hair bulb, as well as cells contributing to the IRS. FGF22 signals predominantly through FGFR2b, often requiring Klotho coreceptors for optimal binding and activation in keratinocytes. The primary downstream pathway activated is the RAS/MAPK/ERK cascade [97,98]. The concerted action of FGF22 on these molecular targets results in the proper hardening of the hair shaft cortex and cuticle, the formation of a fully keratinized IRS that molds the hair shaft, and ultimately the production of a mature, structurally sound hair fiber ready for sustained growth during the remainder of anagen.

During the anagen-to-catagen transition, FGF22 expression shifts, and its signaling targets the dermal papilla and potentially the epithelial stem cell niche. Crucially, it signals back to the epithelial keratinocytes surrounding the DP and in the regressing epithelial strand. Its mechanism involves actively suppressing survival signals and promoting apoptotic pathways. Recent in vivo studies using conditional *Fgf22* knockout mice show significantly delayed catagen onset and reduced caspase-3 activation in the regressing follicle [69,99,100].

In summary, FGF22 exhibits a remarkable stage-specific duality: in late anagen, it acts through FGFR2b/MAPK to drive terminal differentiation and keratinization in precortex/cortex/IRS cells, ensuring hair shaft maturation. At the cycle’s end, it becomes a key catagen inducer by promoting apoptosis, suppressing Wnt/β-catenin and Shh survival pathways, and synergizing potently with TGF-β2 to trigger and accelerate the destructive remodeling of the follicle [101].

### 5.2. FGFs That Are Found During Growth and Inhibit Hair Growth

FGF5 acts as a crucial inhibitory factor for hair elongation, predominantly expressed in the late growth phase of hair follicles, where it serves as a key regulator that transitions hair follicles from the growth to the degenerative phase. Studies have identified FGFR1, a high-affinity receptor for FGF5, expressed primarily in DPCs, hair progenitor cells, and hair follicle stem cells. In particular, the ORS cells of the hair follicles produce FGF5, which then inhibits the proliferation of these cells in a paracrine manner, stopping the growth phase and initiating degeneration [66,102]. Furthermore, FGF5 adversely affects DPS-induced hair development and regeneration. At a concentration of 20 ng/mL, recombinant human FGF5 (rhFGF5) enhances the expression of BMP4 in DPS, suppressing the accumulation of β-catenin and inhibiting Wnt signaling [103]. Western blot analysis reveals that treatment with FGF5 downregulates β-catenin expression by 1.8-fold while upregulating both BMP4 and Wnt5a by 1.5-fold, suggesting that rhFGF5 impedes hair growth and regeneration by promoting BMP signaling [104]. Additionally, the knockdown of the *Fgf5* gene in animals such as mice, goats, and rabbits inhibits the conversion of arachidonic acid to epoxyeicosatrienoic acids and dihydroxyeicosatrienoic acids via the activation of the PI3K/AKT signaling pathway. This increases amino acid (AA) content in skin tissue, thus promoting cashmere goat growth [105]. In addition, Zhang’s research revealed that the crosstalk between androgen and Wnt/β-catenin signaling plays a major role in the increase in wool and active hair-follicle density due to the activation of c-MYC and KRTs associated with inner root sheath (IRS) in *FGF5-* knockout (KO) sheep, and the Shh pathway is also involved in this process as a downstream pathway of Wnt/β-catenin signaling [106].

## 6. Recent Research on FGFs Detected During Telogen

### 6.1. FGFs That Are Found During Telogen and Promote Hair Growth

FGF1, FGF2, and FGF13 are detectable during the telogen phase and stimulate hair growth. FGF1, also known as acidic FGF (aFGF) or HBGF, has been linked to cashmere yield, fineness, and length in Inner Mongolia cashmere goats in a genome-wide association study that also highlighted the *semaphorin 3D (SEMA3D)*, *envoplakin (EVPL)*, and *SRY-box transcription factor 5 (SOX5*) genes [107]. FGF2, commonly known as basic FGF (bFGF), demonstrated promising results in a Phase I clinical trial that evaluated the effects of intradermal injections in patients with androgenetic alopecia [108]. The trial reported that 86% of the participants showed a decrease in fine hairs, while 80% exhibited an increase in terminal hairs. Furthermore, the average diameter of the hair shaft increased by 3.03 μm after eight treatments, confirming the safety and efficacy of the therapy [66]. In a separate study, four AGA patients treated biweekly with a non-exfoliative dot matrix laser and a topical application of a compound growth factor solution containing FGF2 showed improved hair regeneration and density without significant adverse reactions [65]. Additionally, in vitro experiments with DPCs demonstrate that FGF2 enhances DPC lineage differentiation, prevents spheroid contraction, and preserves versican expression. Synergistic effects on DPC-related gene expression were observed when FGF2 and platelet-derived growth factor-AA (PDGF-AA) were combined in a half-diluted CAO (CAO 1/2) medium, essential for promoting DP-specific characteristics under 3D culture conditions [109]. According to existing research, FGF2 activates MAPK for follicle development [38]. FGF2 and FGF21 upregulate MAPK signaling in secondary hair follicle development, as observed in cashmere goats. This pathway coordinates proliferation and differentiation during follicle cycling [38].

FGF13 expression is elevated in the enlarged portion of hair follicles, where stem cells reside primarily. These stem cells oscillate between active and dormant states, suggesting a potential role for FGF13 in the self-renewal of hair follicles. FGF13 levels are higher during the growth phase, decrease in the degeneration phase, and increase again in the quiescent and long-term regeneration phases. This pattern suggests that FGF13 may facilitate the transition of hair follicles from the quiescent phase to the regenerative phase, thereby promoting hair follicle growth. A plausible mechanism is that enhanced FGF13 signaling during the quiescent phase surpasses the activation threshold of hair follicle stem cells, triggering their transition into long-term regeneration [51,110]. FGF13 plays an intracellular role with proper MAPK signaling, but recent studies also suggest an extracellular interaction that triggers a downstream signal cascade. FGF13 is expressed across diverse vertebrate organisms and plays a myriad of cell- and tissue-specific roles in both development and organ homeostasis [110].

### 6.2. FGFs That Are Found During Telogen and Inhibit Hair Growth

FGF18 is predominantly expressed during the resting phase of hair follicles, where its primary role is to maintain the follicles in this state and prevent their transition to the growth phase. In mice, a subcutaneous injection of FGF18 at the end of the hair follicle growth stage immediately stops progenitor cell proliferation and significantly suppresses growth [111]. Forkhead box protein P1 (Foxp1), another crucial factor, maintains hair in the resting phase. The functional loss of Foxp1 stimulates cellular activation and abbreviates the resting stage, while its overexpression inhibits cell proliferation and induces a hair follicle cycle arrest. Importantly, *Fgf18* acts as a key downstream target gene of Foxp1, and the application of exogenous *Fgf18* can rectify premature activation of hair follicles in knockout mice [112]. Furthermore, FGF18 inhibits keratinocyte proliferation without triggering terminal differentiation, strengthening its regulatory role in hair follicle dynamics [113]. Reduced expression of *Fgf18* and *Bmp6*, the factors involved in HFSC quiescence, was observed in the HFSC niche of the hairpoor mouse. In addition, disturbed expression of Wnt signaling molecules, including *Wnt7b*, *Wnt10b*, and *Sfrp1,* was observed, which induced the telogen-to-anagen transition of HFs in the hairpoor mouse [114].

## 7. Recent Research on FGFs Detected in All Periods

FGF9, a heparin-binding growth factor (HBGF), binds predominantly to FGFR2 and FGFR3 and requires the formation of a tripartite complex involving heparin, receptor, and ligand. Recent findings have shown its role in hair growth. In studies involving mice, the administration of FGF9 significantly accelerated new hair growth after hair removal, thus shortening the growth phase [5,115]. The researchers employed safflower as a biological reactor to produce the FGF9 protein, and the resulting transgenic safflower oil body, when applied to the hairless backs of C57BL/6 mice, resulted in noticeably thicker hair and a higher count of new hairs compared to controls, with an rhFHF9 concentration of 60 g/L [115]. In another study, variations in *FGF9* expression were observed throughout the hair follicle cycle, influencing wool growth [116]. Sheep treated with *FGF9* in their DPCs demonstrated increased proliferation rate, cell cycle progression, and decreased mRNA and protein levels of the Wnt/β-catenin signaling pathway marker gene *catenin beta-1* (*CTNNB1*). In contrast, DPCs with *FGF9* knockdown exhibited the opposite effects. Thus, FGF9 improves DPC proliferation and cell cycle and regulates hair follicle growth and development via the Wnt/β-catenin signaling pathway [116,117]. What’s more, recombinant human FGF9 promotes hair growth in mice by modulating the TGF-β/BMP/Smad signaling pathway. This pathway is critical for hair follicle cycle transitions, particularly in inducing the anagen phase [5].

## 8. Regulatory Roles of Other FGFs in the Hair Follicle Cycle

Overactivation of FGF8 signaling has been shown to disrupt the expression of crucial genes for hair follicle development, such as *Shh* and *Bmp4*, ultimately impeding development. This phenomenon was observed in mice that overexpressed FGF8 in their epidermis. Excessive FGF8 not only inhibits epidermal cell proliferation but also promotes apoptosis, thereby obstructing hair follicle development [118]. Additionally, FGF8 expression is reduced in dermo-specific conditioned *Blimp 1* knockout mice, illustrating its critical role in normal follicular function [119]. Although the exact contribution of FGF8 to identity specialization remains unclear, its presence in type I hair cells indicates that identity is established earlier than previously believed, occurring shortly after terminal mitosis. The unique cell-specific expression of *Fgf8* makes these modified cell lines invaluable for targeted regeneration strategies, indicating their potential as a significant resource in developmental studies [120].

FGF20, a member of the FGF9 subfamily, plays a crucial role in various stages of hair growth, mediating its effects through pathways such as Wnt and Bmp4. rhFGF20 has been shown to induce ciliary follicle growth in a dose-dependent manner in mouse ciliary follicle organ cultures. However, high concentrations of rhFGF20 paradoxically inhibit their growth, indicating that rhFGF20 promotes hair stromal cell proliferation and activates the Wnt/β-catenin signaling pathway [121]. Additionally, FGF20 is instrumental in dermal condensation of hair follicles. Advanced imaging techniques, including 3D and 4D, have revealed that FGF20 regulates cell cycle exit and directs cell migration during dermal condensation in primary hair follicles of mouse skin. The absence of FGF20 results in a failure of dermal condensation in protective and many secondary hairs, as well as basal plate invagination, ultimately leading to hairlessness [122]. In embryos with stable expression of epithelial β-catenin but lacking FGF20, mesenchymal condensation ablation was observed, demonstrating the essential role of FGF20 in hair development. Moreover, significant external root sheath protrusions beneath the sebaceous glands are detectable during the quiescent phase, indicating active responses [123]. These protrusions are absent in subsequent growth cycles. These findings suggest that FGF20 can activate hair follicle stem cells and induce the transition of hair follicles from the quiescent to the growth phase.

FGF21, a recently identified member of the FGF family, significantly affects the cycle and structure of hair follicles. Research involving *Fgf21* knockout mice (*Fgf21−/−*) has shown a significant reduction in hair growth rate and smaller hair follicle diameters compared to their wild-type counterparts, suggesting a vital role for *Fgf21* in hair follicle development [124]. These knockout mice also show decreased hair regeneration rates and reduced hair shafts compared to wild-type mice [123]. *Fgf21* induces the expression and phosphorylation of ERK and Akt proteins, thus influencing a complex signaling network that regulates hair growth. These findings indicate that FGF21 plays a crucial role in hair follicle development and hair growth through modulation of the ERK and AKT signaling pathways [125].

**Figure 4:**
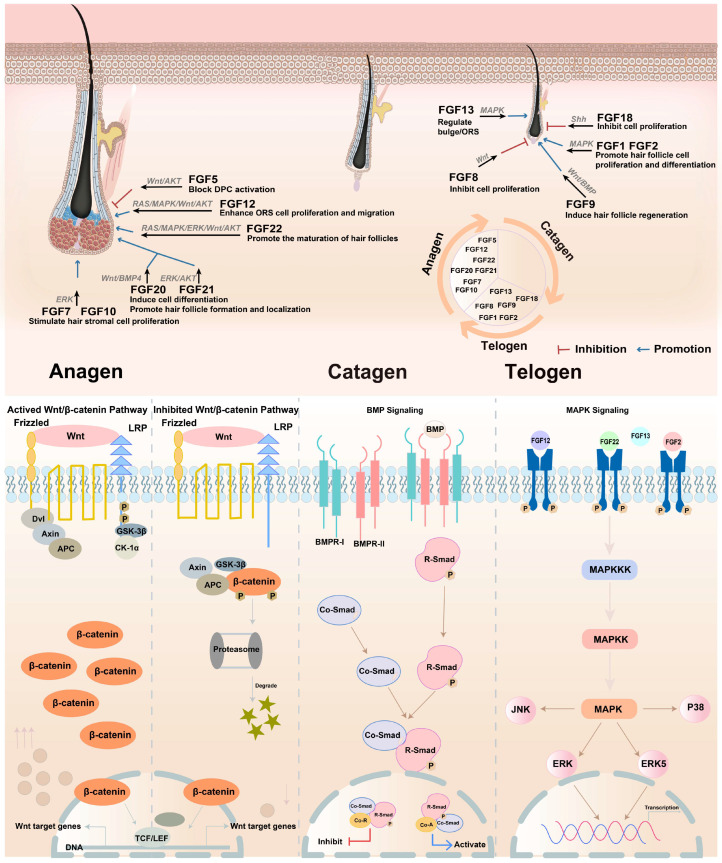
The regulated effects of FGFs in hair follicle growth. In the hair follicle cycle, different FGFs contribute to different effects that promote or inhibit hair follicle growth.

The role of FGF in the hair follicle cycle is concluded in Figure 4. This schematic summarizes the diverse and critical roles played by various FGFs in regulating distinct phases of the hair follicle cycle (telogen, anagen, catagen) and specific cellular compartments. It illustrates how individual FGFs exert precise effects, ranging from promoting proliferation and differentiation (e.g., FGF1, FGF2, FGF9, FGF10, FGF12), follicle formation/maturation (e.g., FGF7, FGF10, FGF22), and regeneration (FGF9), to inhibiting proliferation (e.g., FGF5, FGF8, FGF18) or blocking dermal papilla cell activation (FGF5). Key functions include regulating the bulge/outer root sheath (ORS) (FGF13), enhancing ORS proliferation/migration (FGF12), and inducing differentiation (FGF20, FGF21). The figure highlights the complex spatial and temporal FGF signaling network essential for coordinating hair follicle growth, regression, and maintenance.

## 9. Regulation of FGF Signaling and Androgenic Alopecia

Androgenetic alopecia (AGA) is an autosomal dominant disorder characterized by progressive thinning of terminal hairs into intermediate and vellus hairs. This condition involves alterations in the hair cycle, such as a shortened anagen phase and an extended telogen phase, leading to shorter hair lengths and eventual balding. Early-onset AGA is strongly associated with severe coronary artery disease and metabolic syndrome, with a higher prevalence observed in individuals with an elevated body mass index. Recent studies have identified a link between AGA and severe coronavirus disease 2019 (COVID-19) cases in both genders, a phenomenon now called the “Gabrin sign” [95,126,127,128].

AGA was previously believed to be inherited in an autosomal dominant pattern with reduced penetration in women. However, considering the extremely high prevalence of the disease and the increased risk among relatives of severely affected women, both genetic and environmental factors likely play a role [129]. The inheritance of AGA is familial and heritable, but its pattern remains ambiguous, likely to be both polygenic and multifactorial. Multiple genetic susceptibility loci have been identified, including *histone deacetylase 9 (HDAC9)* on chromosome 7p21.1, chromosome 3q26, the *paired box gene 1 (PAX1)/FOXA2* locus on chromosome 20p11, and the *androgen receptor (AR)/EDAR2* locus on the X chromosome. Furthermore, AGA has associations with body mass index, metabolic syndrome, and severe COVID-19 cases [130].

Practitioners have various options to treat AGA, including oral and topical medications, hormonal therapies, nutraceuticals, platelet-rich plasma, exosomes, microneedling, and more invasive techniques such as hair transplantation. However, treating AGA can be particularly challenging due to the variability in patient responses to conventional therapies and an incomplete understanding of its pathogenesis [131,132,133,134].

Treating androgenic alopecia by regulating FGFs is a novel approach identified in recent years. For instance, Gentile and Garcovich reported that adding dihydrotestosterone (DHT) to DPCs from patients with androgenic alopecia decreases the expression of FGF7 within cells while increasing the secretion of both FGF7 and FGF2 [135]. Research on hair follicle growth has revealed that the expression of FGF7 and BMP inhibitors increases during the late resting period of hair follicles. Both FGF7 and BMP are known to regulate the Wnt pathway in hair progenitor cells, suggesting that DHT-stimulated secretion of FGF7 in DPCs interacts with receptors on hair progenitor cells to inhibit the Wnt signaling pathway, ultimately contributing to the development of androgenic alopecia. Furthermore, androgen increases the number of hair follicles in the dormant phase by stimulating FGF2 expression, leading to alopecia. Additional studies highlight that *Fgf5* is a susceptibility gene for male pattern hair loss, and it promotes the transition of hair follicles from the growth phase to the degenerative phase, resulting in hair loss [69,113]. In cultured ORS cells of hair follicles, an increase in vascular endothelial-derived growth factor (VEGF) concentration correlates with a decrease in *Fgf5* mRNA levels, suggesting that VEGF can promote hair follicle growth by suppressing *Fgf5* expression in secondary hair follicle ORS cells [136,137]. FGF5 is a negative regulator that antagonizes Wnt-mediated anagen prolongation by triggering catagen via undefined crosstalk [102].

The dynamic crosstalk between FGF, dihydrotestosterone (DHT), and Wnt pathways represents a critical regulatory nexus in hair follicle homeostasis and pathology. Studies reveal that FGF signaling works in synergy with Wnt pathways during hair morphogenesis and regeneration, while DHT exerts inhibitory effects on Wnt/β-catenin signaling [138,139,140,141,142,143]. Notably, DHT-induced hair loss involves suppression of Wnt/β-catenin signaling through multiple mechanisms, such as via the DHT-PGD axis that enhances GSK-3β-mediated β-catenin degradation [141], downregulating Wnt reporter genes and target expressions [143], and through oxidative stress-mediated pathway inhibition [144,145]. Conversely, FGF signaling demonstrates complex cross-talk with Wnt pathways, while FGF/Wnt synergy promotes progenitor cell fate decisions during development [146].

These mechanistic insights guide several therapeutic strategies, such as simultaneously targeting FGF and Wnt pathways, with Hedgehog/Notch modulation, which shows promise for maintaining follicular cell phenotypes [147]. Combination therapies exploiting FGF-Wnt synergy may overcome DHT resistance in recalcitrant androgenetic alopecia [142,147,148]. Targeted FGF delivery could bypass DHT-induced Wnt suppression by directly amplifying β-catenin-dependent anagen signaling while counteracting FGF5-mediated catagen effects [143,146,149]. Crucially, patient stratification based on individual pathway dominance (e.g., high DHT vs. low Wnt signatures) could guide precision FGF-based interventions, transforming empirical treatment into mechanism-driven regeneration [138,142,148].

## 10. Conclusions and Perspectives

### 10.1. Conclusions

This review has synthesized current knowledge on the pivotal roles of FGF and their cognate receptors in governing hair follicle biology, from embryonic development through cyclic regeneration. Besides, the interplay between FGF signaling and pathways like Wnt/β-catenin, BMP, and TGF-β is emphasized as central to hair follicle homeostasis. Furthermore, the review highlights the therapeutic potential of targeting FGF pathways to treat hair loss conditions such as androgenetic alopecia, offering mechanistic insights superior to conventional treatments.

### 10.2. Perspectives

While the preceding section has established the well-documented and multifaceted roles of Fibroblast Growth Factors (FGFs) in governing critical aspects of hair follicle biology, the precise molecular mechanisms through which these diverse effects are orchestrated remain a focal point of research. It is now essential to delve deeper into the specific intracellular signaling cascades triggered downstream of FGF/FGFR binding. The biological outcomes elicited by FGFs within the hair follicle microenvironment are fundamentally determined by the activation of distinct downstream pathways. Therefore, the following paragraph will systematically dissect the key signaling mechanisms, elucidating how they transduce FGF signals from the cell surface to the nucleus, ultimately regulating gene expression, cell proliferation, survival, migration, and differentiation events crucial for hair follicle development, maintenance, and regeneration.

**Figure 5:**
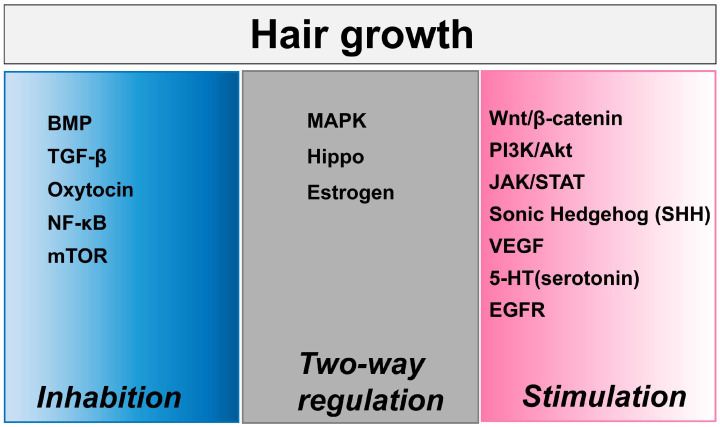
Signaling pathways regulating hair growth. The figure categorizes key molecular pathways under BMP, MAPK, and Wnt/β-catenin signaling hubs, with annotations for inhibitory/stimulatory effects on follicular activity.

Some classical mechanisms regulate hair follicles, including the Wnt/β-catenin pathway, PI3K/Akt and JAK/STAT pathways, BMP pathway, EGF/EGFR pathway, 5-HT (serotonin) signaling, MAPK/Ras pathway, and so on [150,151,152]. The Wnt/β-catenin pathway is one of the most critical pathways for hair follicle development, cycling, and regeneration. Activation of Wnt/β-catenin signaling promotes hair follicle morphogenesis, stem cell activation, and hair shaft elongation, while its inhibition leads to follicle regression. Wnt10b/β-catenin signaling specifically regulates dermal papilla formation and coordinates hair follicle stem cells (HFSCs) with melanocyte activity during regeneration [92,93,148,153,154]. PI3K/Akt and JAK/STAT signaling, which are implicated in cell proliferation and survival, are also activated by certain compounds to enhance DP cell proliferation and hair peg sprouting in organoids [155,156,157,158]. The BMP pathway maintains HFSCs in a quiescent state, counteracting Wnt-driven activation. BMP inhibition is necessary to initiate the anagen phase, highlighting its role in hair cycle regulation [153]. The MAPK/Ras pathway is involved in hair-like sprouting in follicle organoids and secondary hair follicle development, with key genes upregulated during growth [38,159,160]. Shh promotes the proliferation of dermal papilla cells (DPCs) by upregulating downstream genes. During the hair cycle, Shh expression peaks in anagen, primarily localized in the inner/outer root sheath and dermal papilla, driving follicular cell activation [161,162,163]. Figure 5 summarizes critical signaling pathways involved in hair growth regulation; this framework highlights the complexity of molecular crosstalk governing hair growth.

FGFs and FGFRs are integral throughout the various stages of the hair follicle lifecycle, contributing to the intricate balance required for hair regeneration and the cyclical nature of hair growth. FGFs such as FGF7, FGF10, and FGF20 stimulate hair follicle growth by promoting cell proliferation and differentiation. This activation during specific hair cycle phases highlights potential therapeutic targets for enhancing hair regeneration and reversing conditions such as AGA. The ability of FGFs to modulate key signaling pathways such as Wnt and BMP provides mechanistic information on their regulatory functions, providing a foundation for developing targeted hair growth therapies. In contrast, FGFs such as FGF5 and FGF18 are associated with suppression of hair follicle growth, particularly during the transition into the degenerative and quiescent phases of the cycle. Understanding these inhibitory mechanisms offers potential intervention options to delay or alter these phases, especially in conditions characterized by unwanted hair growth or rapid cycles of hair follicles. Table 3 outlines the key findings of FGFs within the hair follicle, as identified in recent studies.

The importance of this review work lies in its comprehensive analysis of hair follicle biology, development, and regeneration techniques, which provides valuable insights for both basic research and clinical applications. By synthesizing current knowledge on signaling pathways and cellular processes, this work establishes a foundation for understanding hair growth mechanisms and developing targeted therapies for various alopecia types.

Regarding techniques for checking hair properties, the review discusses multiple advanced methodologies. Histological examination using hematoxylin and eosin (HE) staining to quantify hair follicle numbers and growth phases [166]. Dermoscopic analysis for quantitative assessment of hair diameter and density improvements [167]. TPEF/SHG imaging techniques for non-invasive evaluation of hair shaft alternation and follicle miniaturization in AGA models [168]. Proteomic approaches (TMT-based) to identify differential protein profiles during hair follicle development [169] and Single-cell RNA sequencing (scRNA-seq) for characterizing hair follicle microenvironments and discovering new cell subpopulations [170]. These techniques collectively enable multidimensional assessment of hair follicle morphology, molecular signatures, and functional states.

Several recent studies have investigated the role of FGF family members in hair follicle regulation. FGF12 was found to promote hair growth by inducing the anagen phase, regulating platelet-derived growth factor CC (PDGF-CC, MDK), and heparin-binding EGF-like growth factor (HB-EGF) expression, and enhancing ORS cell migration [4]. FGF9 was shown to accelerate hair growth via the TGF-β/BMP/Smad pathway in mouse models [5]. FGF5’s role as a negative regulator was further characterized, with mutations leading to prolonged anagen phase [69,102]. Novel FGF5 spliceosomes (FGF5-AS1, AS2, AS3) were identified in rabbit models, showing regulatory effects on dermal papilla cells [164]. FGF21 was demonstrated to be crucial for hair follicle cycling through *CRISPR/Cas9* knockout studies [125]. Additionally, FGF2 incorporated in PLCL/keratin bilayer dressings promoted hair follicle regeneration during wound healing [68]. These studies collectively advance our understanding of FGF-mediated hair follicle regulation. What’s more, convincing insights into the mechanism of FGF function within hair follicles suggest significant therapeutic potential.

Although promising, the clinical application of FGFs presents challenges, including delivery methods, stability, and potential side effects. Advanced formulations and delivery systems, such as encapsulated forms or targeted delivery mechanisms, could enhance the therapeutic potential of FGFs. Clinical trials are necessary to validate these approaches and ensure their efficacy and safety for long-term use. As research progresses, the therapeutic landscape for hair loss and related conditions will likely expand to include more refined FGF-based therapies. Future studies should focus on understanding less explored FGFs, integrating FGF-based treatments with existing therapies to improve efficacy, and developing diagnostic tools for customized treatments based on individual FGF profiles, which could improve therapeutic outcomes and minimize side effects.

## Figures and Tables

**Table 1:** Distribution of FGF mRNAs in different hair follicle growth cycles.

FGF	Sites of mRNA Distribution	Peak Expression
FGF1	Internal root sheath of the hair follicle [52]	Quiescent phase [53]
FGF2	Outer root sheath of the hair follicle, hair parent material [54]	Quiescent phase [54]
FGF5	Outer root sheath of the hair follicle [55]	Growth stage VI [56]
FGF7	Hairy papillae [52]	Growth stage V [57]
FGF10	Hairy papillae, outer root sheath of hair follicles [58]	Growth stage V [59]
FGF13	Hair follicle bulge area [53]	Quiescent phase [60,61]
FGF18	Internal root sheath of the hair follicle, hair follicle bulge area [53]	Quiescent phase [62]
FGF22	Internal root sheath of the hair follicle [63]	Growth stage VI [64]

**Table 2:** Role of fibroblast growth factors in hair follicle growth (+/−).

		Function
FGFs		Action
FGF1	+	Hair follicle differentiation and prevention of radiation-induced apoptosis in hair follicles [4]
FGF2	+	Proliferation of hair follicle cells and prolonging of the growth period [4,68]
FGF5	–	Blocking of DPC activation during the growth phase [69]
FGF7	+	Prolonging of the growth period, proliferation of hair embryo, activation of stem cells [70]
FGF8	–	Inhibition of epidermal cell proliferation [71]
FGF9	+	Induction of hair follicle regeneration after trauma [72]
FGF10	+	Hair follicle formation and morphogenesis [58]
FGF13	+	Regulation of bulge/ORS cells with reduced expression in hyperplasia [73]
FGF18	–	Induction of the rest period and maintenance of stem cells at rest [53]
FGF20	+	Formation of dermal condensate and development of substrate [74]
FGF21	+	Development of secondary hair follicles [75]

**Table 3:** Key advances in FGF roles during hair follicle growth (2020–2025).

Year	FGF Target	Key Finding	Model System
2023	FGF7	Enhances hair shaft growth and elongation in xenograft-cultured hair follicles and improves the survival of hair follicles following chemotherapy and other cytotoxic injuries [76]	Organ-cultured human HFs and scalp skin
2023	FGF10	*MiR-184* within DPCs (SHF-DPCs) influences hair growth by modulating FGF10 levels [83]	Cashmere goat embryos
2022	FGF12	Predominantly expressed in ORS cells, FGF12 shows high expression levels during the growth phase of hair follicles [4]	Mice
2023	FGF22	Dual role: Drives hair shaft keratinization (late anagen) and induces catagen via FGFR2b/MAPK/TGF-β2 synergy [99]	Mice
2024	FGF5	FGF5 alternative spliceosomes inhibit DPC proliferation [164]	Rabbit
2021	FGF1	FGF1 links to yield, fineness, and length of Inner Mongolia cashmere goats [107]	Mongolia cashmere goats
2022	FGF2	Intradermal injections increase terminal hairs (80% patients) and hair shaft diameter (+3.03 μm) in the Phase I trial [108]	AGA patients
2024	FGF13	Facilitate the transition of hair follicles from the quiescent phase to the regenerative phase [110]	Mice
2022	FGF18	A subcutaneous injection of FGF18 at the end of the hair follicle growth stage immediately stops progenitor cell proliferation and significantly suppresses growth [112]	Mice
2023	FGF9	*Fgf9* improves DPC proliferation and cell cycle and regulates hair follicle growth and development via the Wnt/β-catenin signaling pathway [116]	Small-Tailed Han Sheep
2021	FGF8	Excessive FGF8 not only inhibits epidermal cell proliferation but also promotes apoptosis, thereby obstructing hair follicle development [118]	Mice
2024	FGF20	FGF20 secreted from dermal papilla Cells regulates the proliferation and differentiation of hair follicle stem cells [165]	Fine-Wool Sheep
2025	FGF21	*Fgf21* induces the expression and phosphorylation of ERK and Akt proteins, thus influencing a complex signaling network that regulates hair growth [125]	Mice

## Data Availability

No new data were created or analyzed in this study.
